# Rapid utilisation of telehealth services for specialist paediatric general surgery outpatient clinics in response to COVID-19

**DOI:** 10.1186/s43159-022-00214-y

**Published:** 2022-11-23

**Authors:** David Cruise, Haydn Cruise, Michael Collin, Parshotam Gera

**Affiliations:** 1 Department of Paediatric General Surgery, Perth Children’s Hospital, Perth, WA Australia; 2 Department of Emergency Medicine, Fiona Stanley Hospital, Perth, WA Australia

## Abstract

**Background:**

The COVID-19 pandemic is highly infectious and prompted, amongst other changes, strict social distancing requirements for medical practitioners in Western Australia. Despite significant previous research into telehealth, uptake had been limited beyond servicing rural patients, in spite of numerous purported benefits.

**Results:**

Rapid adoption of telehealth for a majority of outpatient appointments was instituted in the sole tertiary paediatric general surgery with high overall success rates — a satisfactory outcome being achieved without requiring an in-person appointment (97.1% for telephone consults, 93.8% for videoconferencing) during the initial COVID-19 crisis from April to June 2020. Success of appointments was lowest for new referrals for undescended testicles at 81.3%. Operations booked through telehealth consultations were only altered in 1 case (5%), and this was not significantly different to in-person bookings (*p* > 0.05). No cases of COVID-19 were incurred by the surgical team or patients during the study period.

**Conclusions:**

We found that with existing technology and minimal training, paediatric surgical consultations were able to be performed via telehealth with high success, high accuracy, and without significant adverse outcomes.

## Background

Severe acute respiratory syndrome coronavirus 2 2019 (COVID-19) is a highly infectious and contagious virus responsible for a global pandemic after the discovery in China in December 2019. Given the ease of transmission, generated from respiratory droplets in asymptomatic or symptomatic patients [1], a common method of limiting cases is the use of social distancing as was adopted in Western Australia with significant success to date. A recent systematic review concluded that social distancing of 1 m was moderately likely to be associated with a large reduction in transmission, the effect increasing with distance [2]. The impact of these restrictions on medical service deployment was significant with reduced patient contact particularly during the height of the crisis. With the strict social distancing measures came a sudden adoption of telehealth to allow outpatient appointments to continue. The use of videoconferencing or telehealth to provide healthcare appointments has been widely studied, although the need to examine patients and to allow better patient to healthcare provider interactions has limited its uptake. Telehealth (or telemedicine) has been defined as the use of electronic information and communication technology to provide and support health care when distance separates participants [3, 4]. In Western Australia, the use of videoconferencing for outpatient clinics was previously utilised for predominantly rural and region consultations [5]. The geographical spread of Western Australia is vast with previous estimates of a median distance of secondary sites — of 317 km [5], meaning that the benefits of telehealth can be significant. A secure videoconferencing platform — healthdirect Video Call service — was available, and utilisation increased rapidly with the COVID-19 crisis to enable patients and their parents to access appointments without special equipment. For many surgical appointments, the view that new or undiagnosed patients must be seen in person to appropriately manage them is often held, despite previous evidence suggesting the viability of telehealth surgical appointments in most situations [6] and previous preoperative diagnostic accuracy of up to 97% and postoperative accuracy of 100% [7].

COVID-19 has been shown to spread rapidly, and healthcare workers are unfortunately significant vectors, particularly to the most vulnerable. In Italy, 20% of healthcare workers were infected, making up 9% of the country’s total confirmed cases at that time [8, 9]. During the recent Victorian second wave, it was found that 70% of healthcare workers who contracted the disease did so through work [10]. Reducing the physical contact through the use of videoconferencing technologies would also help to decrease transmission rates between healthcare workers in whom very high rates of infection can occur. In Australia, and in Western Australia particularly, there have been comparatively fewer infections and deaths compared with other Western nations. The use of social distancing and other policies aimed at preventing COVID-19 via minimisation of contact and transmission, as well as rigorous tracing, was implemented early in an attempt to limit cases. During the peak of cases in Western Australia, elective surgery was restricted to only the most urgent cases or ‘category 1.’

Chuo and colleagues [11] proposed measurement standards in light of the surge in demand seen for paediatric telehealth services with the primary domains including access to care, financial impact/cost, experience and effectiveness, and this correlates with the most prevalent factors identified for choosing telehealth [12]. A previous systematic review found evidence for the enhanced outcomes, improved experience, ease of use and low cost [13]. Potential limitations have also been identified including misdiagnosis, overuse of services, poor rapport or communication, expanded infrastructure costs and overmedication [4, 14]. Recent work in adults has shown more rapid assessment, and perceptions being largely positive by both patients and clinicians with telehealth appointments [15, 16] with 86% of respondents being satisfied, although a higher proportion was noted to prefer face-to-face visits with a surgeon [17]. Other paediatric surgical services have also described their experiences in rapid adoption during COVID-19 with generally positive outcomes and early success [14].

## Methodology

This study took place in the Perth Children’s Hospital, the only tertiary paediatric hospital in Western Australia. Clinical decisions were made by the treating physician per their preference with no interference from the research team. Telephone appointments were made with the clinician through preferred telephony services. Videoconference appointments were conducted by Healthdirect Video Call service with the clinician using a provided computer with a webcam/microphone and the patient utilising their preferred video-capable device (commonly mobile phone, tablet or integrated laptop). Patient visits were identified and clinic letters analysed to ascertain the outcomes. Primary outcome measured was the success of telephone or videoconference with failure being defined as follows: where a satisfactory outcome was not able to be achieved and another appointment was made for the purpose of completing the planned appointment (excluding referral to another medical team, medical specialist or further appointments as routine follow-up). Plans had been made to analyse the numbers of patients and staff found to contract COVID-19; however, the total numbers in Western Australia were minimal, and no known cases related to the general surgical team were found.

## Results

Six-hundred ninety-nine visits were identified and analysed over the 2 months of major distancing restrictions between April and June 2020 after excluding nonattendance and chart reviews. Successful appointments were decided by an independent data collector and verified by the team leader. The average age of included patients was 5.4 years. Analysis of the overall appointments found a high success rate of 97% and 93.8% for telephone and videoconferencing, respectively (Table 1). The higher rate seen with telephone appointments is likely to reflect the inherent bias with more complicated appointments triaged to videoconferencing services. Sub-analysis of the appointments by new (Table 2) and follow-up patients (Table 3) yielded predictable decreases in the number of patients requiring further appointments with only 89–90% of new referrals able to be managed successfully by telehealth appointment, whereas 95–98% were able to be managed for follow-up patients.

**Table 1:** Success rates of appointments

	**Successful**	**Unsuccessful**	**Success rate**
**Face to face**	292	0	100.0%
**Telephone**	203	6	97.1%
**Videoconference**	183	12	93.8%

**Table 2:** Success rates of appointments in new patients

	**Successful**	**Unsuccessful**	**Success rate**
**Face to face**	106	0	100.0%
**Telephone**	27	3	90.0%
**Videoconference**	40	5	88.9%

**Table 3:** Success rates of appointments in follow-up of patients

	**Successful**	**Unsuccessful**	**Success rate**
**Face to face**	186	0	100.0%
**Telephone**	176	3	98.3%
**Videoconference**	143	7	95.3%

**Fig. 1:**
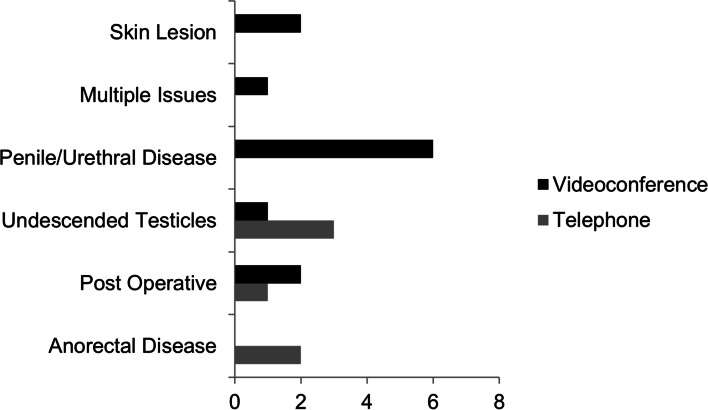
Cause of telehealth appointment failure by diagnostic group

The cause of patient failure in every case was the perceived need to examine the patient in person to clarify a diagnostic dilemma. After categorising the diagnosis of these patients in whom face-to-face follow-up was required (Fig. 1), the two most common groups identified were those of possible undescended testicles and penile or urethral pathology; it should be noted that the latter was a very heterogeneous group only combined for ease of analysis.

For patients referred with undescended testicles, the success rates of first consultations were only 81.3%. This correlates somewhat with previous studies identifying undescended testicles as a group with relatively high difficulty to diagnose accurately via telehealth [18]. Penile or urethral disorders as a group had a 91% success rate, but new referrals had a higher success of 93.1% with the majority of failures being in follow-up patients at 89.5%.

**Fig. 2:**
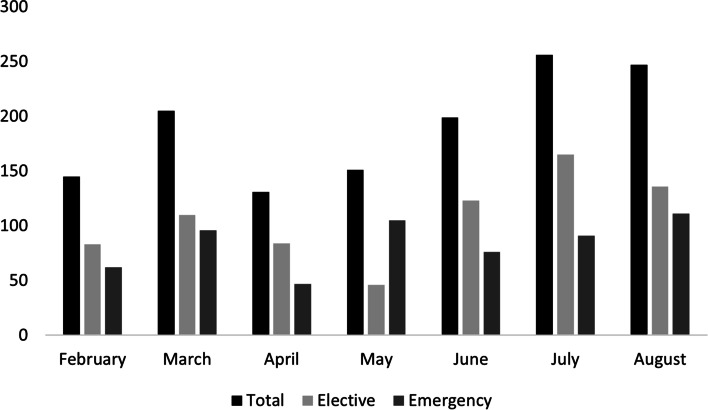
Operative case numbers during study period

Figure 2 shows the operative numbers extending from before and after the period of significant restrictions. Elective surgery numbers decreased during the April and May, during the major restriction period. Elective surgeries increased in the following months.

Notably, zero cases of COVID-19 were found in patients presenting for surgery or surgical staff, and as such, no further analysis was performed.

During the analysis, 71 patients were identified who were booked for surgery after appointments as outlined in Table 4 (49 face to face, 9 telephone and 17 videoconference). Of the 49 patients booked in a face-to-face appointment, 32 underwent the planned procedure, 1 underwent surgery with a procedure added and 16 are awaiting surgery. Of the 9 telephone bookings, 8 underwent as planned, and 1 is awaiting their procedure. Of the 17 booked via videoconference, 12 underwent as planned, 1 had a procedure removed after face-to-face meeting and 4 are awaiting their procedure. No cases were completely cancelled after physical review or an entirely different procedure required. Of face-to-face bookings, 3% were altered compared with 5% of telehealth bookings (*p* > 0.05).

**Table 4:** Patients booked for operative management stratified by appointment type and status

	**New**	**Follow-up**	**Total**
**Face to face**	28	21	49
**Telephone**	3	6	9
**Videoconference**	12	5	17

## Discussion

Our study has looked at a rapid adoption of telehealth services for a state-wide specialist paediatric general surgical service in response to the implementation of social distancing. As such, the results should be interpreted in the context of a service altering the practice suddenly and without a period of adjustment or established procedures. The primary outcome of success of telehealth appointments was encouraging at 94–97%, although as outlined the success of new appointments was less at 89–90% and the vast majority of these failed appointments were elected to be seen in person by the treating specialist. We found that penile/urethral disorders and undescended testicles to be the highest risk of failure of primary telehealth visits, but within these, the undescended testicles were usually in new referrals where the complex nature of deciding surgical management leads to the clinician wanting a preoperative evaluation. The penile and urethral disorders’ failures stemmed mostly from follow-up appointments where concerns about issues were not able to be allayed over telehealth.

It should be noted that like other centres, it has been established practice for patients from regional and rural locations requiring a significant travel to be booked in the day of or before where a high suspicion of surgery was ascertained. Such appointments made after a telehealth consultation were not planned to be included as failures as the ability to triage such patients through telehealth could be considered a strength, though no cases were ultimately found. Of the cases booked for theatre through a telehealth appointment, only the one (5%) was altered, and none was cancelled. It could be suggested that the clinicians opted to conservatively review the patients in person given the lack of familiarity with the technology. Assuming a reasonable accuracy and progression to surgery, particularly if it is able to avoid unnecessary trips for further management, it would not be unreasonable to expand this proportion of patients; however, this would need to be balanced against the risk of underutilising theatre time.

The primary limitation of the study was its non-randomised nature with decisions on whether to review patients in person or via telehealth made by the treating specialist with knowledge of the condition in question. This will have lead to the number of patients posing a greater diagnostic dilemma being allocated straight to the in-person appointments. There were no guidelines provided for making further face-to-face appointments, which may have lead to more patients being recalled than necessary as clinicians not familiar with the potential options may have responded to any uncertainty with in-person assessment. This is likely a result of the sudden need to adopt these services; however, this also provided a real-world perspective in terms of implementing telehealth services without significant prior experience. It was beyond the scope of this project to calculate diagnostic accuracy; however, the high accuracy seen in other studies is encouraging [7].

## Conclusion

Telehealth was able to be instituted with minimal issues given that the appropriate platform was already available. Despite the limited training and education, the success rates for both telephony and videoconferencing-based appointments were high. Our analysis was in line with that of previous work finding that undescended testicles were challenging to assess via telehealth [6] however still had encouraging success rates of 81.3%. Booking of patients for surgery over telehealth was achievable with similar efficacy to in-person bookings. The ability to examine the patient on either the day of surgery or prior provided an adequate safety net with only minor alterations being necessary to a small proportion in our limited series. Our experience suggests that telehealth can be utilised safely and effectively for outpatient appointments for paediatric general surgery patients provided adequate technology is in place and the limitations are appreciated. Expansion of telehealth should be considered by paediatric surgical and medical services, particularly where significant benefits are expected such as the need for social distancing, as long as patient safety and diagnostic accuracy are not compromised.

## Data Availability

The database used for this project will be made freely available to any credible research effort that contacts the primary author of this work.
